# Identification of field caught *Anopheles gambiae *s.s. and *Anopheles arabiensis *by TaqMan single nucleotide polymorphism genotyping

**DOI:** 10.1186/1475-2875-6-23

**Published:** 2007-02-27

**Authors:** Edward D Walker, Alisha R Thibault, Annette P Thelen, Blair A Bullard, Juan Huang, Maurice R Odiere, Nabie M Bayoh, Elizabeth E Wilkins, John M Vulule

**Affiliations:** 1 Department of Microbiology and Molecular Genetics, Michigan State University, East Lansing, MI 48824, USA; 2 Department of Biochemistry, Michigan State University, East Lansing, MI 48824, USA; 3 Centre for Vector Biology and Control Research, Kenya Medical Research Institute, P.O. Box 1578, Kisumu, Kenya; 4 Atlanta Research & Education Foundation, Malaria Research and Reference Reagent Resource Center, Centers for Disease Control and Prevention, 4770 Buford Hwy., Mailstop F-42, Atlanta GA 30341, USA

## Abstract

**Background:**

Identification of *Anopheles gambiae *s.s. and *Anopheles arabiensis *from field-collected *Anopheles gambiae *s.l. is often necessary in basic and applied research, and in operational control programmes. The currently accepted method involves use of standard polymerase chain reaction amplification of ribosomal DNA (rDNA) from the 3' 28S to 5' intergenic spacer region of the genome, and visual confirmation of amplicons of predicted size on agarose gels, after electrophoresis. This report describes development and evaluation of an automated, quantitative PCR method based upon TaqMan™ single nucleotide polymorphism (SNP) genotyping.

**Methods:**

Standard PCR, and TaqMan SNP genotyping with newly designed primers and fluorophore-labeled probes hybridizing to sequences of complementary rDNA specific for either *An. gambiae *s.s. or *An. arabiensis*, were conducted in three experiments involving field-collected *An. gambiae *s.l. from western Kenya, and defined laboratory strains. DNA extraction was from a single leg, sonicated for five minutes in buffer in wells of 96-well PCR plates.

**Results:**

TaqMan SNP genotyping showed a reaction success rate, sensitivity, and species specificity comparable to that of standard PCR. In an extensive field study, only 29 of 3,041 (0.95%) were determined to be hybrids by TaqMan (i.e., having rDNA sequences from both species), however, all but one were *An. arabiensis *by standard PCR, suggesting an acceptably low (ca. 1%) error rate for TaqMan genotyping in mistakenly identifying species hybrids.

**Conclusion:**

TaqMan SNP genotyping proved to be a sensitive and rapid method for identification of *An. gambiae *s.l. and *An. arabiensis*, with a high success rate, specific results, and congruence with the standard PCR method.

## Background

*Anopheles gambiae *sensu lato (s.l.) is a species complex composed of seven biological species, including two widely distributed and important vectors of human malaria in subSaharan Africa (*An. gambiae *sensu stricto and *Anopheles arabiensis*, whose geographical ranges broadly overlap [1-5]. Two species (*Anopheles melas *and *Anopheles merus*) are restricted to brackish water environments of coastal Africa, and are of regional importance as vectors; the other 3 species (*Anopheles bwambae*, and *Anopheles quadriannulatus *species A and species B) are species of localized distribution [6]. For purposes of basic and operational research and as part of malaria control programmes, it is often necessary to identify field caught *An. gambiae *s.s. and *An. arabiensis*, but they are not distinguishable morphologically. Cytological methods based upon chromosome inversion patterns were the original method to distinguish the species [3]; other methods have included isoenzyme electrophoresis and cuticular hydrocarbon analysis [7]. Elucidation of ribosomal DNA (rDNA) sequences allowed development of probes and primers, permitting application of molecular based methods for species identification, including DNA-DNA hybridization and polymerase chain reaction [8-10]. Scott et al. [8] aligned ca. 1,000 base pairs (bp) of the *An. gambiae *s.l. intergenic spacer (IGS; GenBank U10135), situated between the 3' 28S and 5'IGS regions of the genome, and from these alignments designed a universal forward primer (i.e., a primer conserved amongst five species in the complex) and reverse primers specific for five of the seven known species. Their use in a standard polymerase chain reaction yields amplicons of species-diagnostic sizes in gel electrophoresis. This method has become the standard tool for species identification in this complex, but it has the drawback when applied to large scale field studies of being laboriously slow. Nucleic acids from each specimen must be extracted, and then a standard PCR reaction carried out on each specimen, followed by gel electrophoresis. For large numbers of individuals, and given an unknown success rate of the reaction, the amount of time required can significantly slow the progress of a study. Often, a subsample of the entire sample is done, and then the proportion of either species is inferred to represent accurately the larger sample [11,12]; in other cases, the entire sample is analysed [13-15]. Rafferty et al. [10] suggested a remedy to speed up processing by standard PCR, based upon the use of a 96-pin bacterial replicator to transfer samples and reagents. Additionally, in practice, the reaction often leads to failures, repeated attempts at PCR with the same specimens, and uncertain interpretations for negative samples [16].

Genotyping single nucleotide polymorphisms (SNPs) as alleles using automated PCR in a quantitative format such as TaqMan™, has become a useful tool in detection of nucleotide sequences in which one or a few nucleotides vary in a genomic nucleotide sequence as in SNPs, and where these variations define alleles or genotypes whose presence, or frequency, in a population is of interest [17-20]. The method requires quantitative PCR to a determined number of amplification cycles, and hybridization of fluorescently-labeled probes binding to the sequences of interest within the amplicon. The 5' nuclease activity of the Taq polymerase liberates the fluorescent label (or "fluorophore") conjugated to the hybridized probe, thereby unquenching the fluorophore, and causing it to fluoresce. Intensity of fluorescence is quantified spectrophotometrically. If two probes designed to hybridize to different nucleotide sequences of alleles operating at the same locus (or, in this application, to sequence variants occurring along the same nucleotide consensus sequence) are labeled with different fluorophores at the 5' end of the sequence, then hybridization will yield fluorescence of one fluorophore or the other. The amount of fluorescence of one fluorophore to the other becomes an X, Y bivariate whose values indicate the likelihood of one, the other, or both genotypes being present in the sample [19,20]. Efficiency of probe hybridization is enhanced by conjugation of minor groove binding ligands to the 3' end of the probe and to the 3' direction of the quencher [18,21]. Statistical algorithms based upon cluster analysis and maximum likelihood estimation provide the basis for classification of these fluorescence values to the correct allele or DNA sequence, referred to in technical jargon as "calling" the alleles [20].

The nucleotide sequences of interest within the IGS region aligned by Scott et al. [8] show sufficient variation for application of the TaqMan genotyping technology for identification of the two major malaria vector species in the *An. gambiae *s.l. species complex, namely *An. arabiensis *and *An. gambiae *s.s. There is insufficient variation for application of the method to the remaining species in the complex, although further research may reveal regions of the rDNA that would be useful in this regard. The purpose was to develop a method of higher throughput, with sensitivity and specificity equal to or greater than that of standard PCR.

## Materials and methods

### Mosquitoes

Adult *An. gambiae *s.l. were sampled from rural sites along the north shore of Lake Victoria, west of the city of Kisumu in western Kenya, as described in Huang et al. [22] and Odiere et al. [15]. Collections included hand catches using a mouth aspirator from houses and from pits dug into the ground, Colombian curtain house-exit samples, indoor pyrethrum spray catches, and from clay pots set out-of-doors. Control DNA was obtained from whole mosquitoes of strains available in laboratory colonies and from the Malaria Research and Reference Reagent Resource Center (MR4), namely *An. gambiae *ZAN/U (MRA-594), a DDT resistant strain originating from Zanzibar; *An. gambiae *KISUMU, originating from western Kenya; *An. gambiae *RSP, a strain with reduced sensitivity to permethrin originating from western Kenya; and *An. arabiensis *KGB (MRA-339). Heterologous DNA was from *Ochlerotatus triseriatus *TOUMEY WOODS strain, originating from Michigan State University; and *Aedes albopictus *courtesy of Dr. Steven Juliano, originating from Florida, USA. Colony conditions were as in Benedict [23] and Huang et al. [22]. Mosquito samples were stored individually in 1.5 ml snap-top Eppendorf tubes at -20°C, and mosquito DNA was prepared for PCR as in Rafferty et al [10], with exceptions as outlined below.

### Plasmid DNA controls

Plasmid control papers were prepared for the identification of *An. gambiae *and *An. arabiensis *when using the protocol of Scott et al. [8], and were used in TaqMan genotyping here as well. The ZAN/U (MRA-594) strain of *An. gambiae *and the KGB (MRA-339) of *An. arabiensis *were used. DNA from individual mosquitoes was prepared for PCR by the method of Rafferty et al. [10]. A portion of the IGS including bases 177–981 (Genbank U10135) was amplified from both species using primers 5'-CCTAACAACCCTCTGAGATCC-3' and 5'-CATGCACAAGACATCCTACTACC-3'. PCR reactions consisted of 1 U of *Taq *DNA polymerase (Promega), 0.3 mM MgCl_2_, primers at 1 μM each, 0.08 mM dNTPs, and the polymerase manufacturer's suggested buffer in 25 μl total volume. PCR was performed using a Bio-Rad iCycler (Bio-Rad Life Sciences Research, Hercules, California, USA) using the following conditions: 95°C 5 m, 30 cycles (95°C 30 s, 50°C 30 s, 72°C 30 s), 72°C 5 m. The 805 bp fragment was cloned using the pGEM T-Easy Vector kit (Promega, Madison, Wisconsin, USA) according to the manufacturer's recommendations and transformed into JM109 cells. Clones were sequenced and matched those of Genbank U10135 (*An. gambiae*) and AF470100 (*An. arabiensis*). Plasmids were purified using the Qiagen-tip 500 according to the manufacturer's instructions. Strips of Schliecher & Schuell 903 cards (Whatman, Florham Park, New Jersey, USA) were dipped in water containing 0.5 ng/μl each of both plasmids, allowed to dry overnight, and stored over silica gel at room temperature. Paper spots were punched using a Harris Micro Punch 2.0 mm (Whatman) and placed directly in the PCR reaction mix as needed.

### Conditions for standard and quantitative TaqMan PCR

Individual mosquito specimens from field collections or laboratory strains were prepared for identification by removing a leg with sterile forceps and placing it into one well of a 96-well PCR tray (P/N 951020389 Brinkmann Instruments, Inc., Westbury, New York, USA). Each well contained 40 μl of TE buffer (10 mM Tris-HCl/1 mM EDTA pH 8.0). Trays were covered securely with sterile adhesive foil and placed on water in a sonicator bath (Bransonic ultrasonic cleaner, Shelton, Connecticut, USA) for 5 min. For some samples, 1 μl was taken for conventional PCR using the method discussed in Scott et. al. [8]. All conventional PCR reactions were performed using the Epicentre FailSafe PCR System (Epicentre Biotechnologies, Madison, Wisconsin, USA). The conditions for conventional PCR consisted of 25 μl of 2X Premix E, 1 μl (20 ng) of universal forward primer (5' GTGTGCCCCTTCCTCGATGT), 1 μl (12 ng) of *An. gambiae *s.s. specific reverse primer (5' CTGGTTTGGTCGGCACGTTT), 1 μl (20 ng) of *An. arabiensis *reverse primer (5' AAGTGTCCTTCTCCATCCTA), 1 μl of Epicentre PCR enzyme mix, and sufficient DNA grade water to raise the reaction volume to 50 μl. The reaction programme had an initial step of 80°C for 1 min, followed by 30 cycles of denaturation at 94°C for 30 sec, annealing at 50°C for 30 sec, and extension at 72°C for 30 sec, with a final extension at 72°C for 4 min. The PCR products were separated by electrophoresis on 2% agarose TBE gels, and stained with ethidium bromide. The amplicons were visualized with an ultraviolet transillumination gel documentation system (AlphaImager 2200, San Leandro, California, USA). The predicted DNA bands on the gel (390 bp for *An. gambiae*, 315 bp for *An. arabiensis*) were compared to a 1 Kb reference ladder.

The original tray containing the remaining mosquito sonicates was processed for TaqMan Genotyping Analysis on an ABI Model 7900 HT workstation (Applied Biosystems, Foster City, California, USA). The reaction used TaqMan mastermix (Applied Biosystems P/N 4304437) and the following newly designed *Anopheles gambiae *s.l. universal primer sequences: (Forward) 5'-GTGAAGCTTGGTGCGTGCT-3' and (Reverse) 5'-GCACGCCGACAAGCTCA-3'. These primers correspond to the 623–641 and 772–788 positions of the 5' end of the intergenic spacer region, respectively. A set of two species-specific TaqMan probes, conjugated to minimum binding groove ligands and a quencher at the 3' end, were synthesized to detect "allele X" (*An. gambiae*): 5'VIC-CGGTATGGAGCGGGACACGTA-3' and "allele Y" (*An. arabiensis*): 5' 6FAM-TAGGATGGAGAAGGACACTTA-3'. These probes correspond to positions 744–764 of the 5' intergenic spacer region, where the consensus sequence is TGGTATGGAGCGGGACACGTA [8]. In this region, *An. gambiae *s.s. has two nucleotide substitutions, at positions 744 and 757, whilst *An. arabiensis *has five substitutions, at positions 745, 747, 754, 755, and 762. There are no insertions or deletions for either species in the 744–764 part of this sequence. Results were expressed as fluorescence intensity, and were displayed on an X-Y bivariate plot. The probability that the probe bound to complementary DNA of the homologous, species-specific amplicon is established with a proprietary algorithm, incorporated into the Applied Biosystems Sequence Detector v1.7 software used here, that invokes a maximum likelihood estimator and cluster analysis [20] to call the alleles. Occasionally, sequences were called manually by visual inspection of the fluorescence values, when automatic detection by system software failed to assign a sequence and an undetermined result was returned.

In the first experiment, a set of known sources of mosquito DNA from laboratory strains as described above, or from field specimens reliably determined previously to species by standard PCR [15], were used along with the plasmid controls discussed above, as well as blank well and internal negative controls. Standard PCR was repeated alongside the TaqMan genotyping method. In the second experiment, a random sample *An. gambiae *s.l. collected from houses in a rural area west of Kisumu, in western Kenya [22] was analysed by TaqMan genotyping and standard PCR. For experiments 1 and 2, the TaqMan genotyping method was analysed for sensitivity using an on-line clinical calculator [24] with the following equation: sensitivity = (number of true positives)/(number of true positives + number of false negatives). The reaction success rate, and comparative sensitivity of TaqMan genotyping to detect a true *An. gambiae *or true *An. arabiensis *sample, was compared by the test of the equality of two percentages [25]. In the third experiment, a sample of 3,041 adult *An. gambiae *s.l. from the Bondo and Kombewa Districts of western Kenya [15] was analysed by TaqMan genotyping to assess the success rate of the reaction, and the proportion of the two species in the study population.

## Results

### Experiment 1

**Figure 1:**
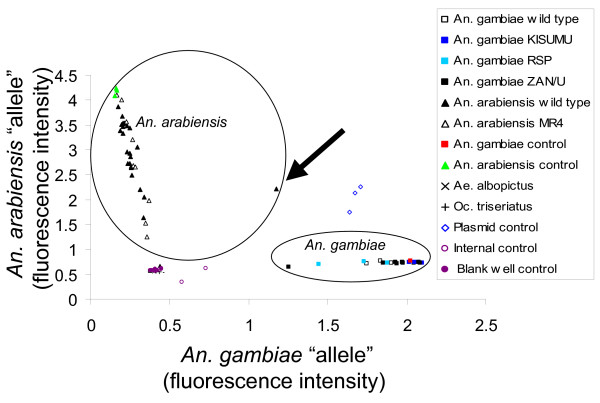
**Bivariate scattergram of relative probe fluorescence from TaqMan genotyping analysis of *An. gambiae *s.s. and *An. arabiensis *s.s**. DNA of various strains and wild types; other DNA sources including *Aedes albopictus *and *Ochlerotatus triseriatus *mosquitoes; plasmids containing rDNA from *An. gambiae *s.s. and *An. arabiensis*; and negative controls. Circles show results of samples from known *An. gambiae *s.s. and *An. arabiensis *sources. The triangle indicated by the arrow, indicating an *An. arabiensis *wild type sample, is an outlier and was classified as a hybrid by TaqMan genotyping.

The bivariate plot of the distribution of fluorescence values of each sample from Experiment 1 is shown in Figure 1. Two of two *Aedes albopictus*, two of two *Ochlerotatus triseriatus*, and all internal controls and blank wells were classified appropriately as "undetermined" in TaqMan genotyping; and all four heterologous mosquito DNA samples yielded no amplicons in standard PCR, as expected. An undetermined result indicates that there was no probe binding to complementary DNA, and, therefore, no amplicon in the quantitative PCR. Three plasmid controls were determined to have both alleles by TaqMan genotyping, as expected, and were positive for both alleles in standard PCR when amplicons were visualized on agarose gels (data not shown). Of 53 *An. gambiae *s.l. from colonies and, therefore, species were known, 51 (96.2% reaction success rate) gave a species identification by TaqMan genotyping. Of these, there were 22 *An. gambiae *s.s. samples of which 21 were determined to be *An. gambiae *s.s. by TaqMan genotyping (95% success rate), and all 21 were positive in standard PCR for *An. gambiae *as well (1.0 sensitivity). Of 31 *An. arabiensis *samples, 30 (97% success rate) were assigned to *An. arabiensis *by TaqMan, and 27 (87% success rate) showed the amplicon typical of *An. arabiensis *in standard PCR. The other four *An. arabiensis *samples were negative by standard PCR. A single sample was called both species by TaqMan genotyping, but it showed a typical *An. arabiensis *amplicon in standard PCR. TaqMan genotyping did not misclassify any samples of known species to the wrong species, giving a specificity of 1.0 for *An. gambiae *and 0.97 for *An. arabiensis*, considering in the latter case that one wild caught specimen was assigned to both species but confirmed by standard PCR to be *An. arabiensis*. A test of equality of percentages showed that there was no difference in success rate of the TaqMan genotyping procedure between *An. gambiae *s.s. (95%) and *An. arabiensis *(97%) (t = 0.24, P > 0.05). Nor was there any difference in the sensitivity of TaqMan in identifying the two species (t = 1.23, P > 0.05).

### Experiment 2

Of the 99 wild caught, female *An. gambiae *s.l. from Huang et al. [22], a total of 96 were analysed by standard PCR and TaqMan genotyping in Experiment 2. Negative and positive controls were normal. Of these 96, ten were *An. arabiensis *by standard PCR and 9 of these 10 were classified as *An. arabiensis *by TaqMan genotyping (sensitivity, 0.9). The tenth specimen was undetermined by TaqMan, giving a success rate of 90% for *An. arabiensis *for this method. Of the remainder, 80 specimens were determined to be *An. gambiae *s.s. by standard PCR and 77 of these were classified as *An. gambiae *s.s. by TaqMan genotyping (sensitivity, 0.96), two of 80 were undetermined, and one of 80 was classified as both species. Of the remaining six samples, three were classified as both species by both methods, one was classified as both species by standard PCR but as *An. arabiensis *by TaqMan genotyping, one yielded no amplicon in standard PCR but was classified as *An. gambiae *s.s. by TaqMan genotyping, and one specimen yielded no result by either method. Sensitivity for TaqMan genotyping for *An. arabiensis *was equivalent to the success rate, and was 0.90. Sensitivity for *An. gambiae *s.s. was 0.95, and was slightly less than the success rate, considering that one specimen was negative by standard PCR but was classified as *An. gambiae *s.s. by TaqMan genotyping. A test of equality of percentages showed that there was no difference in sensitivity of the TaqMan genotyping procedure between *An. gambiae *(96%) and *An. arabiensis *(90%) (t = 0.72, P > 0.05) when compared to standard PCR.

### Experiment 3

**Figure 2:**
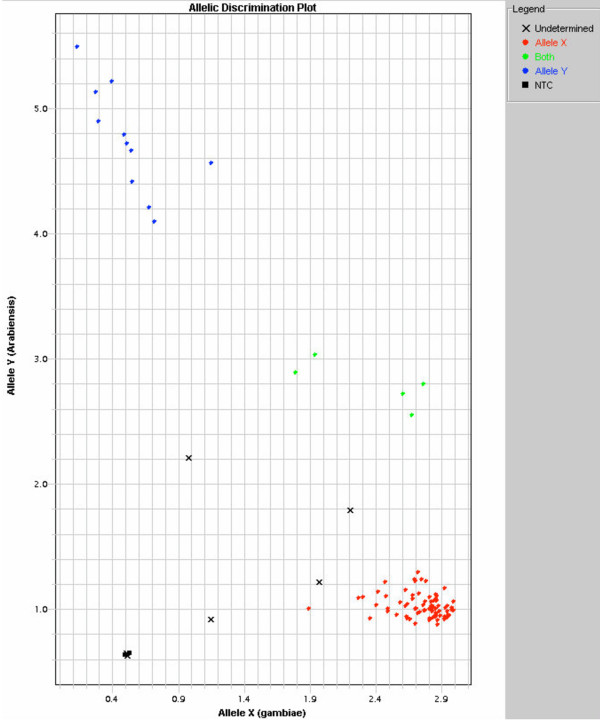
**Bivariate plot generated from TaqMan genotyping, given as output from the ABI Prism 7900HT Sequence Detection System (proprietary hardware and software from Applied Biosystems)**. Samples are from wild caught *An. gambiae *s.l. adults from western Kenya. Red dots are *An. gambiae *s.s., blue dots are *An. arabiensis*, green dots are hybrids (two samples) or plasmid controls (three samples), black x's are undetermined samples, and black squares are internal negative controls.

Of 3,041 field caught *An. gambiae *s.l. analysed by TaqMan genotyping, there were 2,621 successful classifications, 391 undetermined specimens, and 29 specimens classified as both species, giving a success rate of 87.02% when counting the classifications of "both" as a negative result. There were 1,223 males and 1,398 females amongst the successful reactions, of which 804 males and 766 females were *An. gambiae *s.s., and the remainder were *An. arabiensis*. Overall, the composition of the community was 51.63% *An. gambiae *s.s., and 48.37% *An. arabiensis*. Output as a bivariate plot from the classification system is shown in Figure 2.

**Figure 3:**
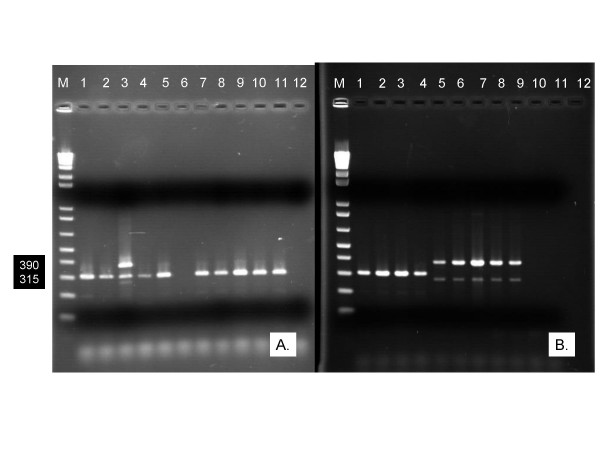
**A. Gel phenotype of standard PCR for wild type *An. gambiae s.l. *from western Kenya, designated as hybrids by TaqMan genotyping**. Lanes 1 – 11, field samples; Lane 12, a negative control. Lane 3 shows a true field hybrid with bands at the predicted 390 and 315 bp positions; lane 6 was a nonreactive sample in standard PCR. All other lanes are *An. arabiensis *by standard PCR, showing a predicted 315 bp product. **B. Gel phenotype of standard PCR for wild type *An. arabiensis *and *An. gambiae *from western Kenya**. Lanes 1 – 4, *An. arabiensis *by TaqMan genotyping. Lanes 5 – 9, *An. gambiae *by TaqMan genotyping. Lanes 10 – 12, undetermined by TaqMan genotyping, and standard PCR yielded no amplicon. A 390 bp amplicon is predicted for *An. gambiae *and a 315 bp amplicon is predicted for *An. arabiensis*. Lane M in both figures is a 1 Kb molecular weight ladder.

Because TaqMan genotyping experiments yielded 29 specimens classified as both *An. gambiae *and *An. arabiensis*, standard PCR was done on eleven of them, all females. Results showed that nine were *An. arabiensis *by gel phenotype, having a 315 bp amplicon, one sample did not react, and one sample had 315 and 360 bp amplicons, indicating a hybrid of both species or at least, both amplicons were present (Figure 3A). Additionally, the following samples were processed by standard PCR: four extractions classified as *An. arabiensis*, five classified as *An. gambiae*, and three classified as "undetermined" by TaqMan genotyping (Figure 3B). Of these, all of the successful classifications matched the standard PCR result, and further the 3 undetermined samples yielded no amplicons in standard PCR either.

## Discussion

The results of the three experiments reported here demonstrate that the TaqMan genotyping system provided a high reaction success rate and acceptable sensitivity in identification of *An. gambiae *s.s. and *An. arabiensis *from field caught *An. gambiae *s.l. adults in western Kenya. Because the rDNA sequence information used to develop the primers and probes was based upon that used for standard PCR for *An. gambiae *s.l. throughout Africa [8], it is very likely that the method would apply to other specimens from other geographic areas. The fact that no reactions occurred with a sample of culicine mosquitoes suggests that the method is specific as well. The reactions here all involved a single leg of each specimen, from which DNA was extracted with use brief use of a common sonicator bath, leaving the remainder of the specimen for analysis of other attributes, such as parasite infection, parity determination, body size measurement, etc.

The results from experiment 1 and 2 indicated a relatively higher success rate than in experiment 3; an explanation for this difference may be related to the handling of a much larger number of specimens in the latter study. Occasionally, it was observed that legs of individual mosquitoes would adhere to the side of the wells of the PCR tray during sonication, just above the 40 ul volume of extraction buffer; and thus DNA would not have been extracted in those few cases. The most parsimonious explanation for the slightly decreased reaction success rate in experiment 3 is this form of systematic operator error, and unlikely to be any aspect of the quantitative PCR or genotyping procedures thereafter. Indeed, in experiments 1 and 2, where fewer specimens were tested and they were handled more carefully, success rate was higher, ranging from 90 to 97%. Others have considered the issue of poor DNA quality of field-collected and archived samples, and have suggested a restriction fragment length polymorphism approach to species identification [26,27], or have not observed it to be a problem [10]. The TaqMan method here obviates the need for several steps that normally are required in standard PCR, including multiple sample transfers; use of a standard thermal cycler; and preparing, visualizing, interpreting agarose gels in gel electrophoresis, and disposal of ethidium bromide-contaminated waste.

The proportions of the two species in the field populations agree with other studies in western Kenya, where these two species commonly overlap in geographic distribution and larvae occur in the same habitats [11,15,16,28]. Results of experiment 2, in which *An. arabiensis *was about 10% of the population of *An. gambiae*, were in complete agreement with those of Huang et al. [22], from which study those specimens came. Thus, the TaqMan genotyping method appears to repeat well the standard PCR method when archived specimens are retrieved, although standard PCR was repeated here on those samples for purposes of comparison, in a blinded fashion. Of interest was that a small number of specimens were determined to be both species in this study. For example, in experiment 3, twenty-nine of 3,041 or 0.9% of individuals were called hybrids. It cannot safely be concluded that these were true hybrids, vs. an artifact of laboratory contamination leading to DNA from the two species ending up in a single extraction; these were field collections that were handled and sorted in multiple ways until they were ultimately processed as described here. Nonetheless, others have suggested that genes introgress from one species to the other [29], and indeed *An. arabiensis*/*An gambiae *s.s. hybrids have been observed in nature but at a very low rate (ca. 0.1%) [30]. When 11 of those 29 classified as hybrids by TaqMan genotyping from experiment 3 were then processed by standard PCR, only one showed both amplicons in the gel phenotype, suggesting a true hybrid; or possibly, it represented a sample with contaminating DNA. It was a female, which (in contrast with males) could have both alleles such that hybrids could be detected with this method [30]. The remainder of the females was *An. arabiensis *in standard PCR, which result suggests that the TaqMan genotyping method may err slightly in a false positive direction for both alleles when the true species is *An. arabiensis*. The 18 other specimens were males, and thus a hybrid result from them must have been from contaminating DNA. Nonetheless, the error rate was less than 1% and quite acceptable.

## Authors' contributions

EDW designed the experiments, analysed and interpreted data, as well as drafted and revised the manuscript. ART, APT, and BAB carried out the experiments and data collection, and JH participated in mosquito collection, identification, and data collection. EEW prepared the plasmid controls. MRO, MNB, and EDW participated in mosquito collection, and JMV provided institutional support for this study.
